# Assessing Housing Accessibility for Older Adults in Japan: A Content Validity Study

**DOI:** 10.1093/geroni/igaa057.357

**Published:** 2020-12-16

**Authors:** Rumiko Tsuchiya-Ito, Björn Slaug, Tomonori Sano, Miki Tajima, Sakiko Itoh, Kazuaki Uda, Takashi Yamanaka, Susanne Iwarsson

**Affiliations:** 1 Dia Foundation for Research on Ageing Societies, Tokyo, Japan; 2 Lund University, Lund, Sweden; 3 Waseda University, Saitama, Japan; 4 Tokyo Medical and Dental University, Tokyo, Japan; 5 The University of Tokyo, Tokyo, Japan

## Abstract

Scientifically validated tools to assess housing accessibility for older adults in Japan have been lacking. To address this, a rigorous procedure of adapting an existing housing assessment tool—the Housing Enabler, developed in Sweden—for valid use in Japan was conducted. The original tool was translated into the Japanese language, using established translation procedures. In the process, researchers checked the appropriateness of technical terms and adjusted specifications to be in accordance with Japanese standards. An expert panel approach was used to validate the content of the Japanese Housing Enabler. Thirteen certified occupational therapists, architects and care-managers (average experience=14.5 years) participated as experts in the content validity study. They rated each item with regard to relevance for assessing housing accessibility in Japan, on a scale from 1(=Not relevant) to 4(= Highly relevant). They suggested adjustments and additions that they found to be relevant to capture particularities of Japanese housing and building design. After individual ratings, the experts gathered for consensus discussions on suggested revisions of the item list. As a result, the number of items was substantially increased (from 161 to 283). A content validity index (CVI) was calculated for each item (i.e., the proportion of experts rating the relevance as at least 3). Using a recommended threshold of CVI ≥0.78, more than 90% of the items were considered relevant, thus supporting the content validity. However, the large amount of items might jeopardize the feasibility of the instrument. Further studies are needed to evaluate feasibility, criterion-related validity and aspects of reliability.

